# Reproducibility of Central Corneal Thickness Measurements in Normal Eyes Using the Zeiss Cirrus 5000 HD-OCT and Pentacam HR

**DOI:** 10.2174/1874364101812010072

**Published:** 2018-05-18

**Authors:** Elmira Baghdasaryan, Xiwen Huang, Kenneth M. Marion, Tudor C. Tepelus, Homayoun Bagherinia, SriniVas R. Sadda, Hugo Y. Hsu

**Affiliations:** 1 Doheny Eye Institute, Los Angeles, CA, USA; 2 Department of Ophthalmology, David Geffen School of Medicine, UCLA, Los Angeles, CA, USA; 3 Carl Zeiss Meditec, Inc., Dublin, CA, USA

**Keywords:** Cornea, Central corneal thickness, Imaging, Optical coherency tomography, Pachymetry, Scheimpflug technology

## Abstract

**Objectives:**

To determine the repeatability and reproducibility of Central Corneal Thickness (CCT) measurements using two different anterior segment imaging modalities, including those obtained with the new anterior segment lens attachments for the Cirrus 5000 HD-OCT.

**Methods:**

A total of 32 eyes from 16 normal volunteers (8 male, 8 female) were enrolled in this prospective study. CCT was measured by the same examiner using the Cirrus 5000 HD-OCT and Pentacam HR. The results of CCT obtained by each method were averaged and compared using t-test analysis. The agreement between the measurement methods was evaluated. Coefficient of Repeatability (CoR) and Intra-Class Correlation Coefficient (ICC) were computed.

**Results:**

The mean measurements taken with the Cirrus OCT anterior chamber lens (CCT_AC_), HD cornea lens (CCT_HDC_) and pachymetry scans (CCT_Pach_) were 545.35 ± 31.02, 537.87 ± 26.82, and 532.04 ± 29.82 µm, respectively. The mean CCT obtained with the Pentacam (CCT_Pent_) was 545.51 ± 30.71 µm. CCT_Pent_ were significantly higher than CCT_HDC_ and CCT_Pach_ (*p*< 0.0001). In contrast, the CCT_Pent_ and CCT_AC_ were similar (*p*=0.87). CCT, as evaluated by the two different instruments, showed excellent correlation (*r* > 0.98, *p*< 0.0001) with an ICC > 0.99 (95% CI, 0.97 – 0.99). CoR was the highest for CCT_Pach_ (3.7 ± 1.4, 95% CI (3.0- 4.6)).

**Conclusion:**

CCT measurements from the Cirrus OCT using the new anterior segment lens attachments and the Pentacam HR are highly correlated. This should allow the use of a standardized correction factor if necessary to inter-relate the measurements between the two devices.

## 1 INTRODUCTION

Accurate measurement of Central Corneal Thickness (CCT) is critical for diagnosing corneal diseases, such as keratoconus and Fuchs Endothelial Corneal Dystrophy (FECD), as well as for monitoring corneal endothelial cell function [1-3]. Evaluation of CCT is also an essential parameter for refractive surgery in order to mitigate the possibility of iatrogenic corneal ectasia development [4]. CCT analysis is also useful for accurate glaucoma diagnosis and management, as it is well known that there is a 0.35-0.38 mm Hg increase in intraocular pressure (IOP) for every 10 µm increase in CCT [5-9]. With the introduction of the collagen cross-linking treatment for keratoconus, the evaluation of CCT has gained further importance as values higher than 400 µm after epithelial debridement are deemed essential for protecting the corneal endothelium from the deleterious effects of ultraviolet-A radiation [10]. CCT evaluation has also been used as a parameter for corneal morphology assessment in contact lens wearers [11].

Both contact and non-contact devices using different techniques or strategies for assessment are currently available to clinicians for CCT estimation. An understanding of the underlying principles for a specific imaging technology is essential for accurate data interpretation, especially since non-contact CCT measurement devices are potentially more practical to employ in a busy clinical practice.

Ultrasonic Pachymetry (USP) is a contact method and has been traditionally regarded as the gold standard for CCT evaluation [12-15]. The contact nature of the USP method introduces patient discomfort and may increase the risk of infection and corneal epithelial damage. The USP approach also has several potential pitfalls which could result in erroneous diagnoses. Foremost, the reliability of USP depends on operator skill and technique, including the requirement for perpendicular placement of the probe with respect to the cornea [16]. Misinterpretation of the results can also occur after instillation of topical anesthesia which produces epithelial edema and CCT overestimation [17]. Also, displacement of the tear film with the probe has been shown to result in CCT underestimation; therefore, non-contact methods have become preferable in current clinic practices [18].

Current non-contact technologies for CCT evaluation include specular microscopy, scanning slit-beam topography, Scheimpflug technology-based cameras, and Optical Coherence Tomography (OCT). These imaging techniques have several advantages in addition to their non-contact nature of application: ease of use, high-resolution imaging, mapping functions, and comparability/correlation with the gold standard USP [19, 20].

Fewer studies, however, have evaluated the accuracy and repeatability of different CCT measurement methods [12, 20, 21]. The Pentacam HR (Oculus, Wetzlar, Germany) is a non-contact rotating Scheimpflug technology that has been shown to be repeatable and reproducible for CCT measurements [19, 22, 23]. Schiempflug based systems use rotating cameras and reconstruct the three-dimensional structure of the cornea from two-dimensional optical sections, which provide sharp images with detailed analysis from the anterior corneal surface through to the posterior aspect of the crystalline lens [24]. The Pentacam (Oculus Inc.), one of the commercially available Schiempflug instruments, is available in three models: Basic, Classic and High Resolution (HR). This system integrates two digital cameras for both the pupil tracking and image capture from the anterior segment. The HR rotating Scheimpflug technology used by the Pentacam allows cross-sectional imaging of the cornea by a 1.45 megapixel camera that rotates along the optical axis from 0 to 360 degrees and records 138.000 true elevation points within seconds. It uses a 475nm wavelength blue Light-Emitting Diode (LED) to provide anterior and posterior surface topography of the cornea, pachymetry, anterior chamber angle, depth and volume data as well as crystalline lens analysis (densitometry). The instrument-based software allows automatic analysis of various anterior segment parameters and takes 25 images per measurement within 2 seconds.

In contrast, the Zeiss Cirrus 5000 HD-OCT (Carl Zeiss Meditec, Dublin, CA, USA) is based on Spectral Domain(SD) OCT technology that uses coherence inferometry and measures the delay of back-reflected light. The device takes up 27,000 A-scans per second and has an axial resolution of 5 µm [25]. It evaluates both retinal and anterior segment structures. SD OCT operates 65 times faster than its predecessor Time Domain (TD) OCT devices. Two external Anterior Segment (AS) lenses (cornea and anterior chamber) have recently become available to facilitate measurement of CCT, irido-corneal angles, angle-to-angle distances, anterior chamber dimensions, and crystalline lens vault. There are few studies, however, comparing CCT measurements obtained by these two different non-contact devices [26-28], particularly since the introduction of these new AS-OCT lenses, which have not been validated yet.

In this study, we take CCT measurements from the Zeiss OCT with the new AS lenses and compare them with a Scheimpflug camera to determine the repeatability and reproducibility of the OCT CCT measurements. To the best of our knowledge, this is the first study to evaluate these new AS lenses and to compare them with the results from a Scheimpflug camera.

## 2 MATERIALS AND METHODS

This prospective study was conducted at the Doheny Eye Center of UCLA in Pasadena, California. The study was approved by the Institutional Review Board of the University of California Los Angeles and conducted in accordance with the ethical standards stated in the Declaration of Helsinki and in compliance with the regulations set forth by the Health Insurance Portability and Accountability Act. Written informed consent was obtained from all subjects. All the participants were volunteers from the Doheny Eye Institute, who had recent eye exams establishing their eligibility for this study.

The exclusion criteria included: age < 18 years, active ocular pathology, corneal pathology, any history of ocular surgery or trauma, recent contact lens wear (within 1 month), systemic diseases with ocular involvement, and astigmatism > 2 Diopters (D). CCT readings were obtained from both eyes for each volunteer using the Zeiss Cirrus 5000 HD-OCT (Carl Zeiss Meditec, Dublin, CA, USA) with two AS lenses (Anterior Chamber (AC) and cornea) and the Pentacam HR (Oculus, Wetzlar, Germany) instruments. The sequence of measurements with the Pentacam HR and the Zeiss Cirrus HD-OCT was randomly chosen. All measurements were taken by a single examiner after 14:00 to minimize the effect of diurnal variations on CCT readings [29]. No topical anesthesia or lubricating eye drops were used in this study. For optimal scan quality, the volunteers were asked to blink twice before each measurement to form a smooth tear film on the cornea. Two CCT measurements were obtained for each study eye per scan type and recorded for subsequent statistical analyses.

### 2.1 Imaging Devices and Measurement Technique

**Fig. (1):**
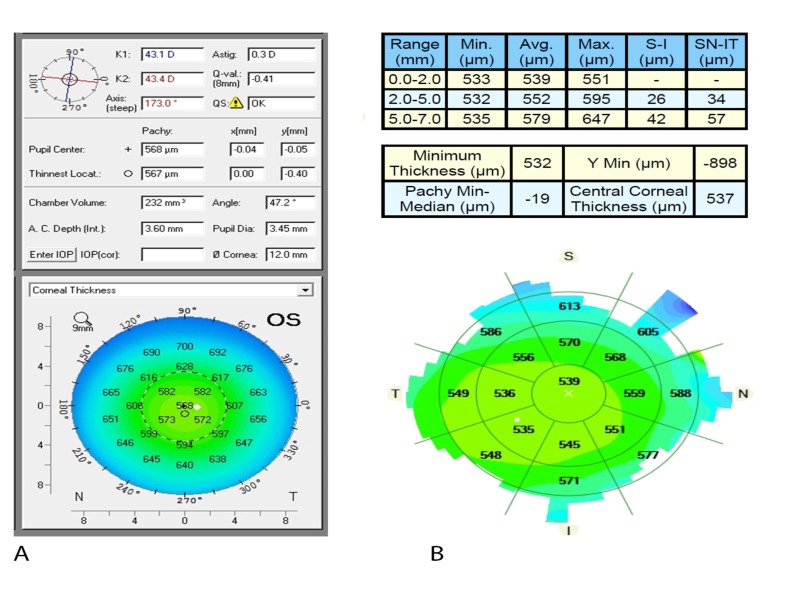
Representative images of the Pentacam HR (**A**) and pachymetry scan reports derived from the Zeiss Cirrus HD-OCT (**B**).

The Pentacam HR was employed to take 2 successive scans for each subject’s eye by a single examiner in one session. There was a short break between acquisitions to eliminate measurement interdependence. Apex pachymetry readings were recorded, averaged and used for subsequent CCT analysis. Images were automatically taken as soon as the Schiempflug camera was centered on the corneal apex at the pupil plane. Before each measurement, subjects were instructed to blink to create an optically smooth tear film over the cornea, and then to hold their eyes open during the image acquisition process. All measurements taken from scans with an examination quality specification of “OK” were considered valid and used for statistical analyses (Fig. **1A**).

For the Zeiss Cirrus 5000 HD-OCT, high-resolution images were taken with both new external Cirrus AS lenses: the cornea-specific lens and the AC specific lens. The cornea lens attachment was used for the pachymetry map and HD cornea scans. The AC lens was used to obtain an AC scan. The Cirrus CCT was basically measured using both methods: manually with the help of the built-in calipers (HD cornea) and automated method (pachymetry map scan, AC scan). Each external lens was mounted on the OCT device with the help of a magnet for AS imaging. The HD cornea scan generates a single scan with a scan depth of 2 mm and a length of 9 mm as specified by the manufacturer. The caliper tool offered with the HD cornea scan was employed for manual measurement of the CCT Fig. (**2B**). It was placed on the central cornea corresponding to the hyper-reflective reflex seen on the scan (*i.e.* at the corneal apex), although it was challenging to place the tool precisely on the corneal apex in the hyper-reflective area.

The pachymetry scan consists of 24 radial B-scan lines (1024 samples per B-scan) with a scan depth of 2 mm. A color-coded thickness map of the cornea was generated after image acquisition and CCT from 0-2 mm sector was selected for subsequent CCT analysis Fig. (**1B**). The thickness was defined as the distance from a point on the anterior corneal surface to the closest point on the posterior corneal surface. The pachymetry analysis tool provided automated cornea thickness measurement in seventeen sectors. Images were captured after the horizontal single scan line was placed on the corneal apex where the hyper-reflective corneal reflex was visible. Repeat scans were taken if the initial scan was decentered or had a poor corneal apex reflection.

The AC scans generated a wide-field image of the front of the eye at the depth of 5.8 mm with A scan length of 15 mm as shown in Fig. (**2A**). The image provides an overall view of the AC with bilateral irido-corneal angles in one glance. The manufacturer’s software provides several automated measurements including CCT in µm, angle-angle distance in mm, AC depth in mm, and lens vault in µm.

### 2.2 Statistical Analysis

All datasets were checked for normal distribution using the Kolmogorov-Smirnov test and analyzed using the R Core Team [30]. Results are presented as the mean ± Standard Deviation (SD). Paired *t*-tests were applied to compare CCT values obtained from the two different devices. The Pearson’s correlation coefficient (*r*) was used to determine the relationship between the measurements of the two instruments (values > 0.7 indicating a strong positive correlation between 2 different devices). Linear regression was used to compare CCT measurements between the two different devices. Intra-operator repeatability was calculated with the two measurements obtained by the single examiner. Coefficient of Repeatability (CoR) and Intra-class Correlation Coefficients (ICC) were also calculated. The CoA was calculated as 1.96 times the standard deviation of the differences in the 2 measurements obtained for each of the two comparisons. The CoA is the value below which the difference between 2 measurements from 2 different devices can be expected to fall with 95% probability. The limits of agreement (LoA) were calculated as the mean difference between the two measurements ± 1.96 times the standard deviation of the differences. Bland – Altman plots were used to assess the reliability of the measurements [31]. *P* values < 0.05 were considered statistically significant.

## 3 RESULTS

### 3.1 Demographics

A total of 32 eyes from 16 normal volunteers (8 male, 8 female) were enrolled. Their average age was 32.3 ± 4.8 years (range: 24-42 years). The mean refractive error derived from the Pentacam HR was 0.7 ± 0.4 diopters (D).

### 3.2 Central Corneal Thickness Measurements

The mean CCT values measured by the Cirrus HD-OCT and Pentacam HR are shown in (Table **1**).

**Fig. (3):**
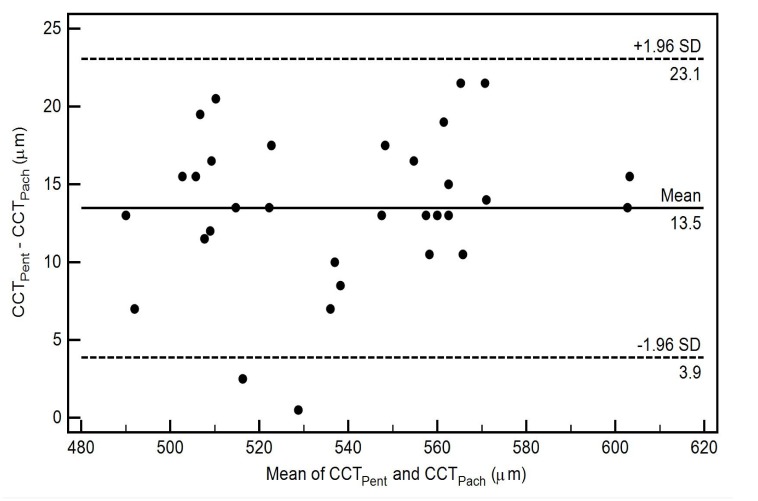
Bland-Altman plot comparing CCT_Pent_ and CCT_Pach_. Mean value, lower and upper limits of agreement are indicated. The mean difference is represented by the solid line and the 95% confidence limits by the dotted lines.

**Fig. (4):**
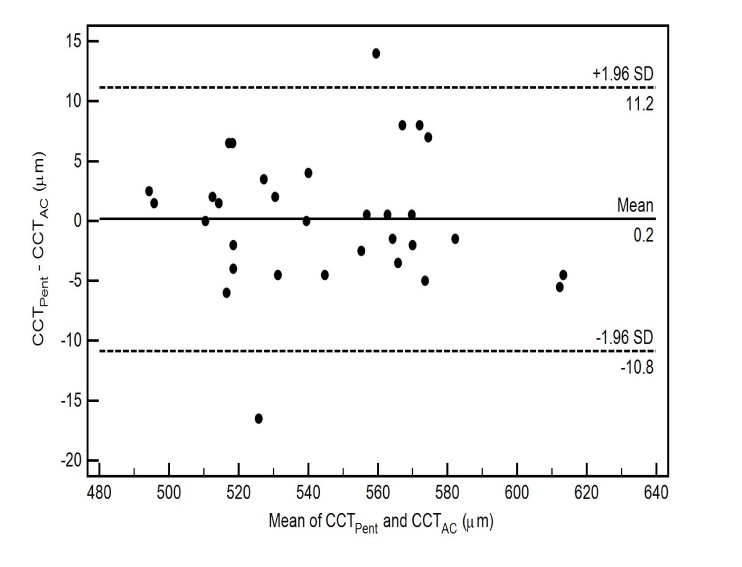
Bland-Altman plot comparing CCT_Pent_ and CCT_AC_. Mean value, lower and upper limits of agreement are indicated. The mean difference is represented by the solid line and the 95% confidence limits by the dotted lines.

**Fig. (5):**
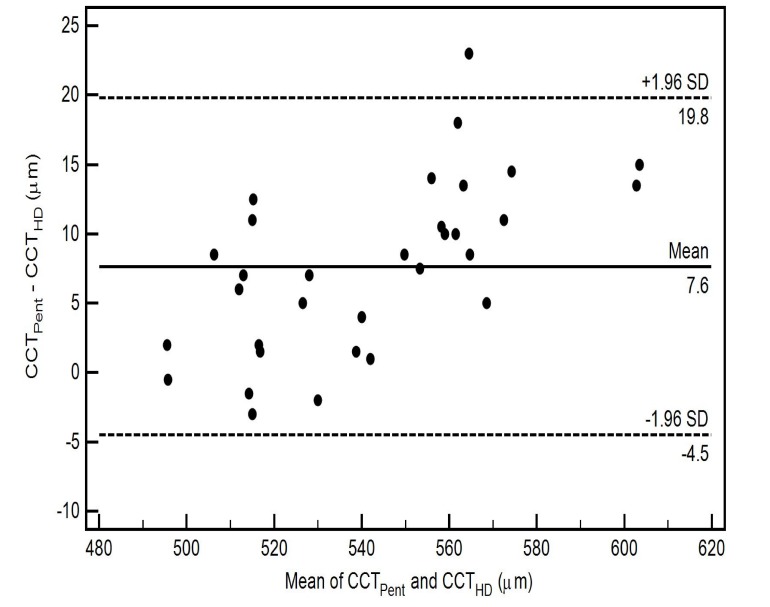
Bland-Altman plot comparing CCT_Pent_ and CCT_HD_. Mean value, lower and upper limits of agreement are indicated. The mean difference is represented by the solid line and the 95% confidence limits by the dotted lines.

The pairwise comparisons of the CCT measurements using 3 different scan types of Cirrus HD-OCT with Pentacam HR in healthy eyes are also shown in Table **2**. All pairwise comparisons demonstrated thinner CCT readings for the Cirrus HD-OCT, nevertheless there was no statistically significant difference between the CCT measurements from the Cirrus HD-OCT AC scan (CCT_AC_) and the Pentacam HR (CCT_Pent_) (*p*-value = 0.87) Table **2**. The level of agreement between the two instruments for each scan type, as well as the mean of the difference between evaluations generated by the two instruments, is illustrated by the Bland-Altman plot (Figs. **3**-**5**).

**Fig. (6):**
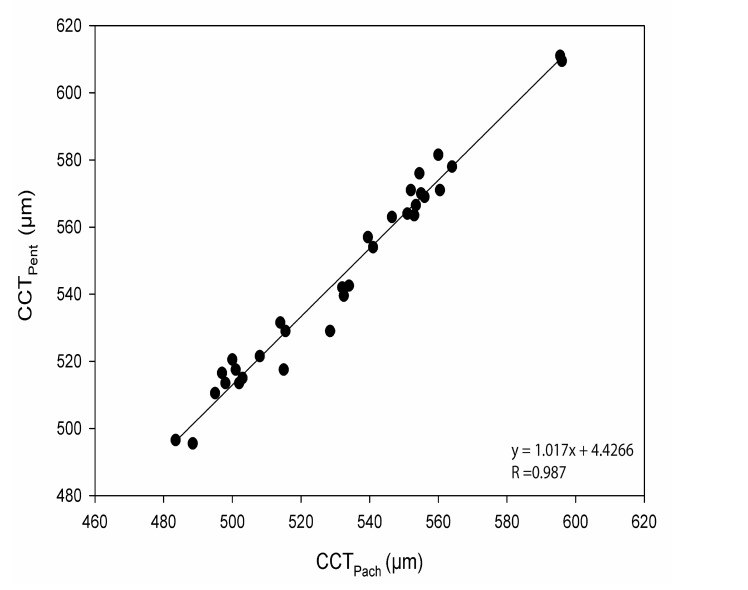
Scatter plots between CCT_Pent_ and CCT_Pach_ measurements (µm) showing a linear regression of *y = 1.017x + 4.42.*

**Fig. (7):**
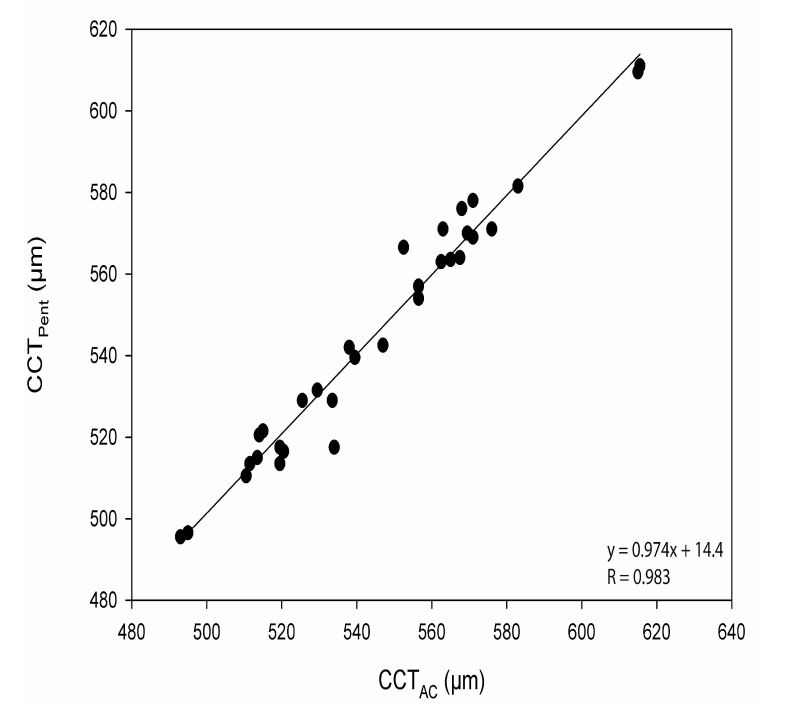
Scatter plots between CCT_Pent_ and CCT_AC_ measurements (µm) showing a linear regression of *y = 0.97x + 14.42.*

**Fig. (8):**
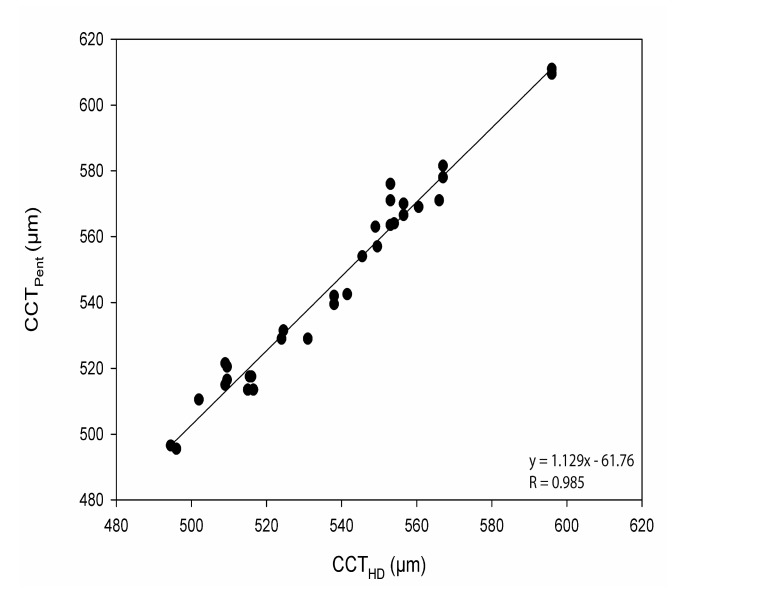
Scatter plots between CCT_Pent_ and CCT_HD_ measurements (µm) showing a linear regression of *y = 1.129x – 61.76.*

The CoA for CCT_Pent_ and Cirrus HD-OCT pachymetry map scan (CCT_Pach_), CCT_Pent_ and CCT_AC_, CCT_Pent_ and Cirrus HD-OCT HD cornea scan (CCT_HDC_) was 9.6, 11.0 and 12.1 µm, respectively. 95% LoA for each pair are shown in Table **2**. The magnitude of the LoA determines if two instruments can be used interchangeably as seen in Figs. (**3**-**5**). Linear regression analysis was used to evaluate the association of variables between devices. The slope of regression lines for CCT_Pach_, CCT_AC_ and CCT_HDC_ against CCT_Pent_ was 1.017, 0.97 and 1.129, respectively (*p* < 0.0001) (Figs. **6**-**8**).

Linear regression analysis, revealed the following relationships: *CCT_Pent_ = 1.017CCT_Pach_ + 4.42; CCT_Pent_ = 0.97CCT_AC_ + 14.42; and CCT_Pent_ = 1.129 CCT_HDC_ – 61.76.* These equations can be used to predict the CCT_Pent_ based on Cirrus HD-OCT measurements of CCT with the specific AS lenses (the cornea and AC). All CCT measurements showed excellent intra-operator repeatability (ICC> 0.99) as shown in Table **2**. The CoR by a single observer was 3.8,12.8, 5.8 and 7.6 for CCT_Pach_, CCT_AC_, CCT_HDC_, CCT_Pent_, respectively.

## 4 DISCUSSION

In this study, we compared non-contact central corneal thickness measurements generated by OCT and Scheimpflug devices in order to assess the level of agreement and reproducibility. Repeatability itself refers to the variation in repeat measurements made on the same subject under identical conditions, meanwhile reproducibility refers to the variation in measurements made on a subject under changing conditions (*e.g.* different measurement methods or instruments being used). Establishing repeatability and reproducibility is critical for confident use of the measurements in clinical practice.

In the present study, we observed that the CCT measured by the Pentacam HR was thicker than that determined by the Cirrus 5000 HD-OCT in healthy eyes. Statistically significant differences were found between CCT_Pent_ and both the CCT_Pach_, and CCT_HDC_, although the difference between CCT_Pent_ and CCT_AC_ was not statistically insignificant. Although previous studies have compared OCT and Scheimpflug-derived CCT measurements [21, 26, 27, 32-35], to our knowledge, ours is the first to assess measurements obtained using the new external lenses for the Cirrus OCT.

Chen *et al* reported that Pentacam HR measurements of the CCT were on average 10.9 µm greater than those obtained from RTVue-100 OCT (Optovue Inc., Fremont, CA, USA) [21]. According to the authors, that difference was small and comparable to the reported diurnal CCT fluctuation. The greatest average difference between the Pentacam HR and OCT CCT in our study was 13.5 µm (CCT_Pent_ - CCT_Pach_) Table **2**, which was not higher than 5% of the mean CCT value for each imaging method Table **1**. Also, the imaging time in our study was chosen accordingly to avoid possible diurnal CCT variation. Chen *et al*. also showed comparable reproducibility, high intra-observer repeatability and a high degree of correlation for both instruments. The authors recommended that the Pentacam HR and RTVue OCT can be used interchangeably for CCT measurements in healthy eyes [21].

Another study by Kanellopoulos *et al*. compared the HR Schiempflug camera Oculyzer 2 (Oculus, Wetzlar, Germany) with the RTVue-100 (Optovue Inc., Fremont, CA, USA) CCT values and found a 12.2 µm statistically significant mean difference between the CCT measurements [36]. The authors suggested that the RTVue OCT appeared to report more accurate, but thinner CCT measurements, than the Oculyzer 2. We also observed that SD-OCT-derived measurements of CCT tended to be thinner than with the Schiempflug camera.

Another study by Ishibazawa *et al*. employed the RTVue-100 (Optovue Inc., Fremont, CA, USA) and Pentacam (Oculus, Wetzlar, Germany) to study the accuracy and repeatability of CCT measurements [27]. They also found a high degree of correlation between methods (*r* = 0.97; *p*-value<0.0001), as well as a high level of repeatability with all methods (ICC 0.97-0.98), similar to our study. With respect to the level of agreement in CCT measurements, they showed that the RTVue-100 underestimated the Pentacam CCT value with a mean difference of 22 µm, which was greater than the difference of 13.5 µm found in our study. Similarly, Gonul *et al*. compared CCT measurements taken with the RTVue-100 (Optovue Inc., Fremont, CA, USA) and Schiempflug camera Sirius (CSO Inc., Firenze, Italy) [32]. Although the mean CCT differences between instruments were not statistically significant and CCT measurements were correlated, the measurements were not considered to be interchangeable in clinical practice because of the wide LoA values.

Yazici *et al*. measured CCT in healthy eyes using the Visante OCT (Carl Zeiss Meditec, Dublin, CA, USA) and Pentacam (Oculus, Wetzlar, Germany). Unlike for SD-OCT, this study showed that the mean CCT measured by the Visante was thinner than the Pentacam CCT values by 20.76 µm (*p*-value < 0.0001); measurements from the two methods, however, were correlated (*r* = 0.88) [33]. In our study, we observed a much higher level of correlation between methods with *r* > 0.98 for all comparisons. Doors *et al*. also showed that the Pentacam significantly overestimated CCT compared to the Visante OCT, by a mean of 19.2 µm [35]. The authors suggested that these devices should not be used interchangeably for CCT measurements in healthy eyes.

Kiraly *et al*. used Cirrus HD-OCT 5-line raster mode as well as Pentacam HR to estimate repeatability and comparability of the CCT measurements [34]. The mean difference between these methods was shown to be 11.44 µm, which was comparable to our maximum reported mean difference of 13.5 µm. Intra-examiner repeatability was high as it was in our study, but the level of agreement in CCT values between devices was insufficient to recommend that they can be used interchangeably, without instituting a correction factor.

The major difference between these previous studies and ours was the use of specialized external lenses for capturing the OCT data as well as the difference between wavelength in different OCT platforms used (Visante represents time domain OCT with 1310nm wavelength). Perhaps the difference between the CCT_Pent_ and CCT_Pach_ could be due to the fact that the Pentacam is expressing a single point measurement, while pachymetry scan from AS-OCT is an average of the central 2 mm area in the cornea. In contrast to this, the measurements obtained by the AC and HD OCT were closer to those obtained with Pentacam as all of them are point measurements taken from the apex. Our findings demonstrated a high level of correlation between scans obtained with the different lenses and excellent intra-observer repeatability. Additionally, through our linear regression analysis, we could generate equations which could be used to convert the OCT-derived measurements of CCT_HDC_ and CCT_Pach_ to values one might expect from the Pentacam. Measurements taken with the AC lens (CCT_AC_) were similar to the Pentacam values and can be implemented without using a standardized correction factor.

## 5 LIMITATIONS

The limitation of our study is the lack of evaluation of the inter-examiner repeatability. Future studies are required to explore the precision of the CCT measurements in eyes after refractive surgery and keratoconus eyes before and after collagen cross-linking treatment.

## CONCLUSION

In conclusion, Cirrus 5000 HD-OCT using cornea and anterior chamber lens attachments for anterior segment imaging and Pentacam HR can be used reliably by an experienced operator in clinical practice for CCT measurements in healthy eyes.

## Figures and Tables

**Fig. (2):**
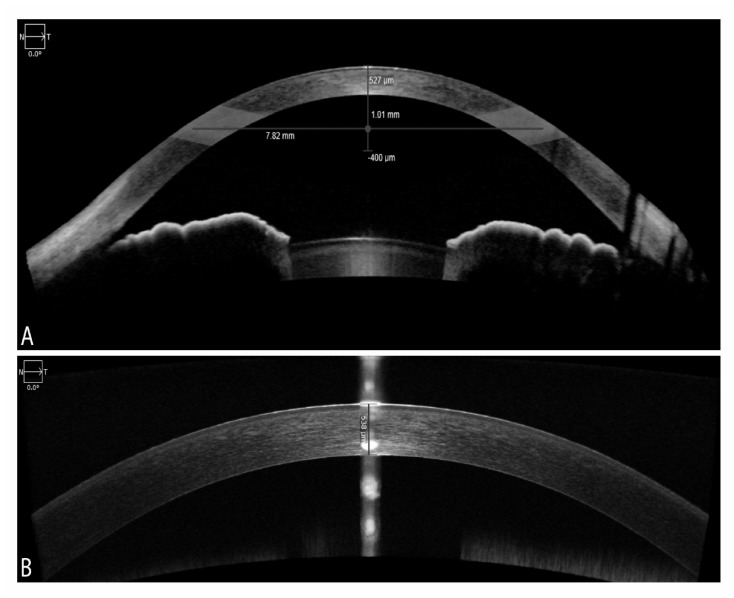
AS-OCT image showing central corneal thickness measurement automated and manual methods by the Zeiss Cirrus OCT AC scan (**A**) and HD cornea scan (**B**), respectively.

**Table 1:** Mean ± SD Values for CCT Measurements in Healthy Eyes Obtained by the Pentacam HR and Cirrus 5000 HD-OCT.

*Parameter*	*Cirrus * *HD cornea*	*Cirrus * *AC scan*	*Cirrus Pachymetry scan*	*Pentacam HR*
CCT (µm)	537.87 ± 26.82	545.35 ± 31.02	532.04 ± 29.82	545.51 ± 30.71

Significant linear positive correlations were observed between the Cirrus HD-OCT and Pentacam HR CCT measurements (Pearson’s correlation, r = 0.98, p-value< 0.0001) (Table **2**).

**Table 2:** CCT Measurement Differences in Healthy Eyes Imaged by the Pentacam HR and Cirrus 5000 HD-OCT.

–	*CCT_Pent_ Versus* *CCT_Pach_*	*CCT_Pent_ Versus* *CCT_AC_*	*CCT_Pent_ Versus* *CCT_HDC_*
**CCT (µm)** **Difference^1^** **(mean ± SD)**	13.46 ± 4.9	0.15 ± 5.6	7.6 ± 6.2
**95% LoA**	3.86 – 23.08	- 10.84 – 11.15	- 4.51 – 19.78
**95% CI**	11.70 – 15.23	- 1.86 – 2.17	5.40 – 9.87
***p* value**	< 0.0001	0.87	< 0.0001
**Pearson Correlation (*r-* value)** **(*p-* value)**	0.987 (CI 0.97-0.99) < 0.0001	0.983 (CI 0.96-0.99) < 0.0001	0.985 (CI 0.97-0.99) < 0.0001
